# Probiotics into outer space: feasibility assessments of encapsulated freeze-dried probiotics during 1 month’s storage on the International Space Station

**DOI:** 10.1038/s41598-018-29094-2

**Published:** 2018-07-16

**Authors:** Takafumi Sakai, Yasuhiro Moteki, Takuya Takahashi, Kan Shida, Mayumi Kiwaki, Yasuhisa Shimakawa, Akihisa Matsui, Osamu Chonan, Kazuya Morikawa, Toshiko Ohta, Hiroshi Ohshima, Satoshi Furukawa

**Affiliations:** 1 Yakult Central Institute, Yakult Honsha Co., Ltd, Tokyo, 186-8650 Japan; 2 Division of Biomedical Science, Faculty of Medicine, University of Tsukuba, Ibaraki, 305-8575 Japan; 3 Japan Aerospace Exploration Agency, Ibaraki, 305-8505 Japan

## Abstract

Suppression of immune function during long spaceflights is an issue that needs to be overcome. The well-established probiotic *Lactobacillus casei* strain Shirota (LcS) could be a promising countermeasure, and we have launched a project to investigate the efficacy of its use on the International Space Station (ISS). As a first step, we developed a specialist probiotic product for space experiments, containing freeze-dried LcS in capsule form (Probiotics Package), and tested its stability through 1 month of storage on the ISS. The temperature inside the ISS ranged from 20.0 to 24.5 °C. The absorbed dose rate of the flight sample was 0.26 mGy/day and the dose equivalent rate was 0.52 mSv/day. The number of live LcS was 1.05 × 10^11^ colony-forming units/g powder (49.5% of the initial value) 6 months after the start of the study; this value was comparable to those in the two ground controls. Profiles of randomly amplified polymorphic DNA, sequence variant frequency, carbohydrate fermentation, reactivity to LcS-specific antibody, and the cytokine-inducing ability of LcS in the flight sample did not differ from those of the ground controls. We can therefore maintain the viability and basic probiotic properties of LcS stored as a Probiotics Package on the ISS.

## Introduction

More than 50 years have passed since the human race leapt into outer space. Thanks to the continuous efforts of astronauts, as well as space scientists, a number of findings regarding the physiology of the human body in space have been reported. Establishment of the International Space Station (ISS) accelerated investigations into physiological responses to prolonged space stays. Accumulating evidence suggests that long-term exposure to the space environment can negatively affect human immune function^1–6^. Weakening of the immune system increases the risk of infectious disease, or even cancer. Because future deep space exploration to the Moon, or to Mars, is a goal, it is essential that we establish measures to combat immune system suppression.

Probiotics are defined by WHO/FAO as ‘Live microorganisms which confer a health benefit on the host when administered in adequate amounts’^7^. Administration of certain probiotic strains modulates immune function and help to provide a good balance of intestinal microbiota, increasing the abundance of beneficial bacteria such as bifidobacteria and lactobacilli and decreasing the abundance of harmful bacteria. Evidence has accumulated that one of these useful probiotics, *Lactobacillus casei* strain Shirota (*L. casei* YIT 9029; LcS) can enhance innate immunity, and in particular can augment natural killer cell activity, probably through the induction of interleukin (IL)-12 production by monocytes/macrophages^8–13^. LcS reaches the intestine alive^14–19^ and balances the intestinal microbiota^19–22^. LcS has a long history of safe use as a food material (over 80 years), and the US Food and Drug Administration has accredited it as Generally Recognized As Safe (GRAS)^23^. Considering its efficacy and safety, LcS has potential for use in combating possible immune problems in crewmembers staying in outer space for long periods of time. To evaluate its efficacy, we have initiated a human space research project in cooperation with astronauts staying on the ISS.

Keeping products containing microorganisms cool is an effective way of extending their shelf life, but one of the challenges in using probiotics on the ISS is the limited use of refrigeration. Moreover, in terms of operational requirements on the ISS, to prevent damage to mechanical devices it is essential to avoid the diffusion of probiotic materials into the microgravity environment. To deal with these challenges, we developed a capsule containing freeze-dried LcS powder (LcS capsule) that is able to keep LcS alive for long periods at ambient temperatures. For the space experiments we also devised a package containing LcS capsules (‘Probiotics Package’) that is specialised to meet the operational requirements of the ISS. The Probiotics Package was subjected to launch into outer space and to stowage on the ISS for 1 month. The Probiotics Package was recovered from the ISS, and we then evaluated the effects of environmental conditions (i.e., temperature and space radiation doses) and the basic probiotic properties of the LcS in comparison with those of ground controls.

## Results

### Configuration of the Probiotics Package

To prevent diffusion from capsules damaged during transportation, including during their launch on the resupply vehicle to the ISS, the LcS capsules were double-packed in a press-through-package (PTP) as the primary package and an aluminium-laminated bag as the secondary package. Desiccants were attached to the inside of the aluminium-laminated bag to prevent absorption of moisture by the LcS powder. A Probiotics Package contained four pieces of PTP sheets, with 40 capsules (10 capsules/sheet) in total.

A temperature data logger and Passive Dosimeter for Lifescience Experiments in Space for bio specimens (Bio PADLES; JAXA, Tsukuba, Japan) were placed in the packages to monitor the environmental temperature and space radiation doses. The validity of the materials and the configuration of the Probiotics Package was reviewed by the Human Space Safety and Mission Assurance Office of the Japan Aerospace Exploration Agency (JAXA) and the Safety and Product Assurance Department of the Japan Manned Space Systems Corporation (Tokyo, Japan), and the packages were approved for stowage on the ISS in advance.

### Environmental conditions during the study period

**Figure 1:**
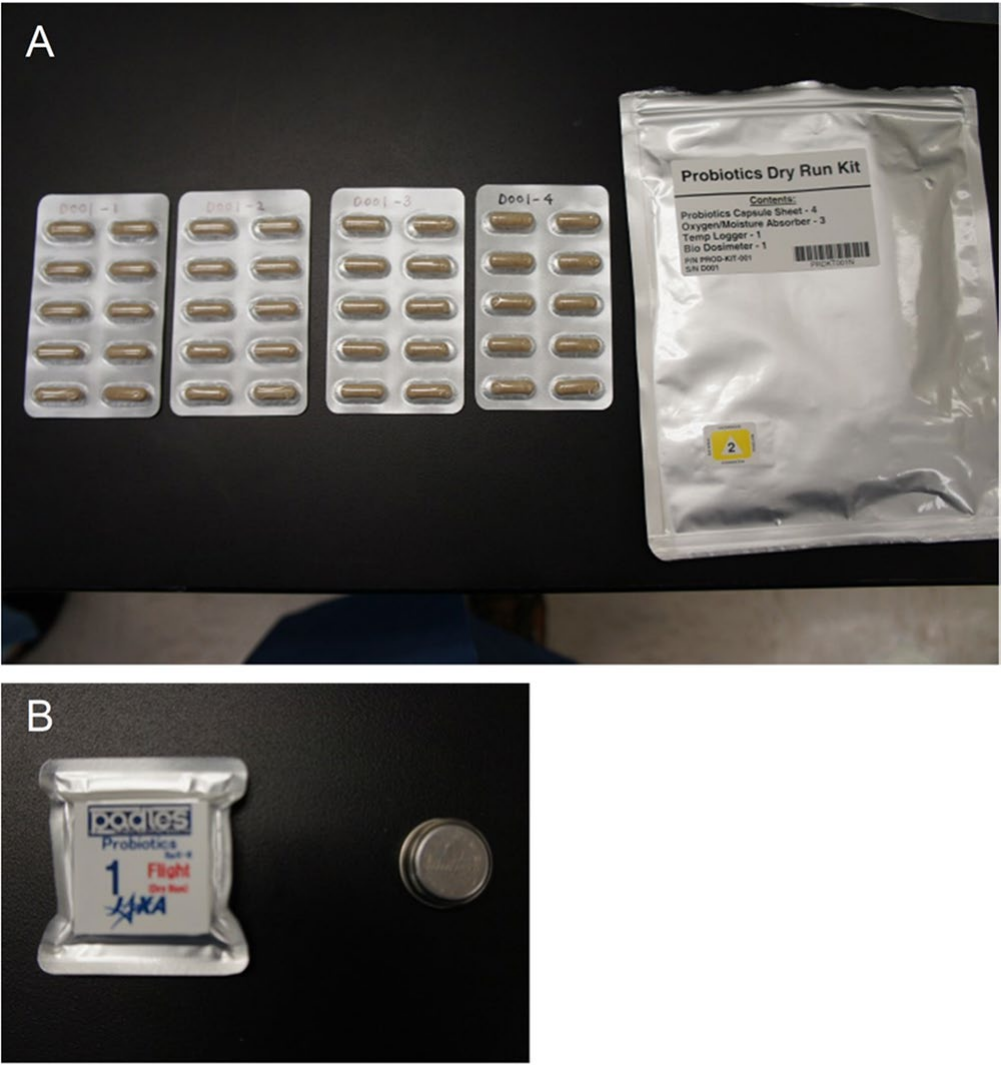
Flight sample capsules in the press-through-package sheets (**A**, left); the outer package (**A**, right); Bio PADLES (**B**, left); and the temperature data logger (**B**, right). Permission to use the digital image of the Bio PADLES including its logo has been made by the Japan Aerospace Exploration Agency.

**Figure 2:**
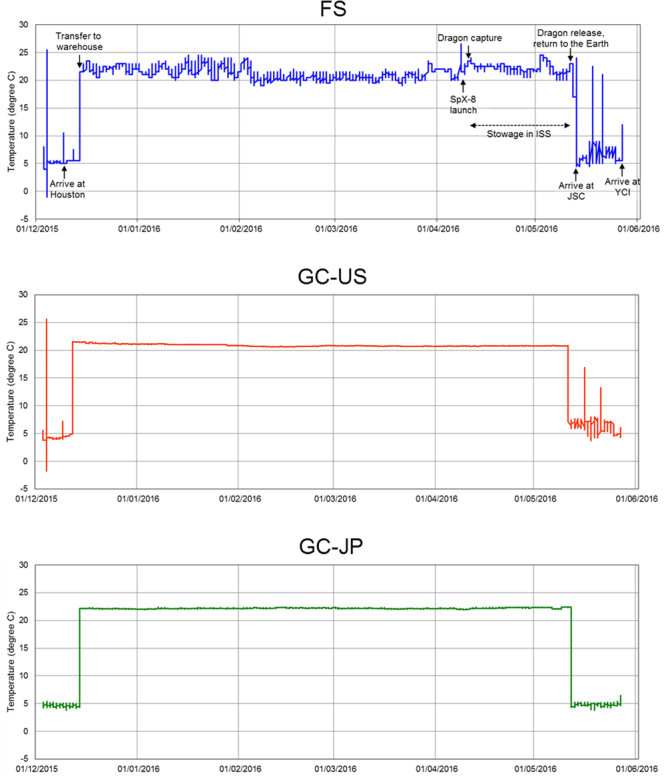
Longitudinal temperature shift of each study product during the study period. The patterns of the flight sample (FS) (**A**) and the two ground controls, GC-US (**B**) and GC-JP (**C**), are shown.

The SpaceX/Dragon spacecraft for the 8th commercial resupply mission (SpX-8) was used for the launch to the ISS and return of the flight sample (FS) of the Probiotics Package. After 1 month of storage on the ISS, the FS was returned to Earth successfully without any damage to the outer package, capsules, temperature data logger, or Bio PADLES (Fig. 1). We evaluated the temperature trends of the FS and the ground controls in the US (GC-US) and Japan (GC-JP) (Fig. 2). The FS was kept at ambient temperature in a range between 19.0 and 24.5 °C from stowage at the warehouse in Houston until return to Earth from the ISS, with an average temperature of 21.5 °C. During stowage on the ISS (between 10 April 2016 and 12 May 2016, Japanese standard time; JST), the temperature ranged between 20.0 and 24.5 °C. GC-US and GC-JP were kept stable at about 21 to 22 °C during the corresponding period. The study products were kept at 4 to 10 °C during other periods (see Materials and methods), except during temporary exposure to ambient temperatures when they were removed from the refrigerators. The dosimetry results for the FS during the 1 month of storage on the ISS are shown in Table 1. Total absorbed dose during stowage on the ISS was 8.53 ± 0.67 mGy in water, and total dose equivalent was 17.08 ± 1.39 mSv. The absorbed dose rate of the FS was calculated at 0.26 ± 0.02 mGy/day and the dose equivalent rate at 0.52 ± 0.04 mSv/day; these values were approximately 130 times those observed in GC-JP (0.002 mGy/day and 0.004 mSv/day, respectively).

**Table 1:** Dosimetry results for the flight sample during storage on the International Space Station.

Total absorbed dose (mGy in water)	8.53 ± 0.67
Total dose equivalent (mSv)	17.08 ± 1.39
Absorbed dose rate (mGy/day)	0.26 ± 0.02
Dose equivalent rate (mSv/day)	0.52 ± 0.04
Mean quality factor	2.00 ± 0.23

Background doses measured in the GC-US sample were subtracted from the yield net doses measured in the flight sample. Errors indicate one standard deviation.

### Survival of LcS during storage

**Figure 3:**
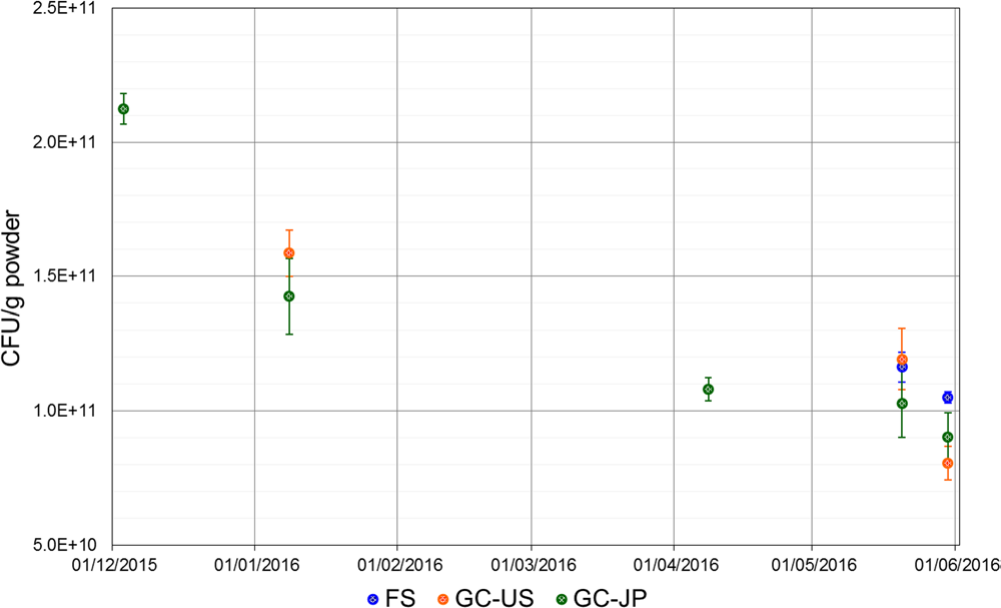
Longitudinal change in the numbers of viable *Lactobacillus casei* strain Shirota (LcS) during the study period. Numbers of viable LcS in the flight sample (FS) (blue) and the two ground controls, GC-US (orange) and GC-JP (green), are shown as CFU/g powder of LcS. Each dot and error bar indicate mean value ± standard deviation, respectively (N = 3).

We evaluated the longitudinal changes in the number of live LcS (colony-forming units; CFU) (Fig. 3). The average number of live LcS was 2.12 × 10^11^ CFU/g powder at baseline (measured soon after packaging of the study product); it gradually decreased during the course of the study, as was also observed in the ground controls. About 6 months after the start of the experiment, the number in the FS had reached 1.05 × 10^11^ CFU/g powder (49.5% of the initial value). The values in GC-US and GC-JP were 8.05 × 10^10^ CFU/g powder (38.0%) and 9.03 × 10^10^ CFU/g powder (42.6%), respectively.

At the end of the study period, the viability of LcS was also evaluated as cell-membrane permeability to propidium monoazide, using quantitative PCR (PMA-qPCR)^24^ and LcS-specific primers. The result for the FS (12.5 ± 0.02 log_10_ cells/g powder, mean ± standard deviation) was equivalent to those of GC-US (12.4 ± 0.08 log_10_ cells/g powder) and GC-JP (12.4 ± 0.10 log_10_ cells/g powder).

### Genetic modification, carbohydrate fermentation and growth profiles

**Figure 4:**
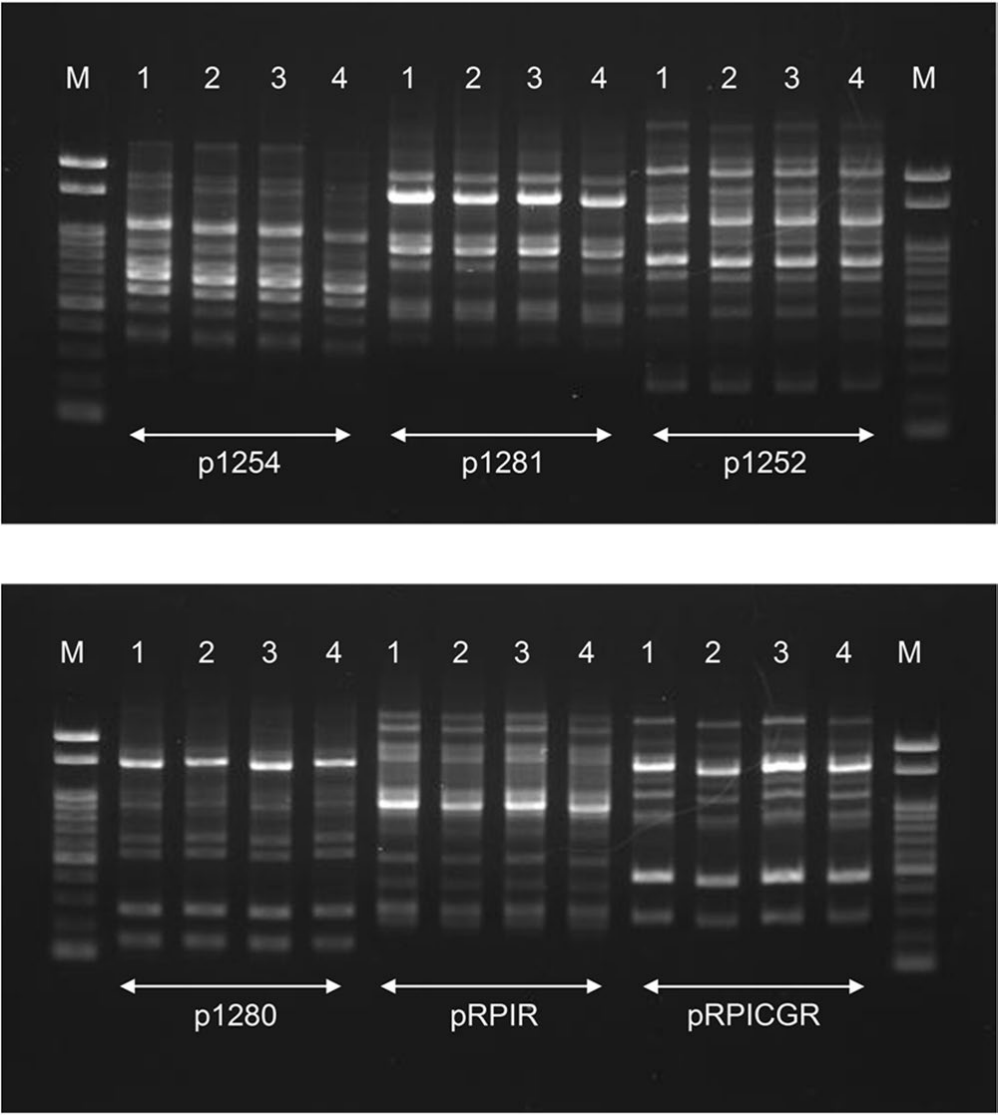
Randomly amplified polymorphic DNA analysis profiles of the study products with six primers. Letters or numbers above each lane indicate: M, molecular weight marker; 1, baseline (using the DNA extracted soon after packaging of the study product); 2, flight sample; 3, GC-US sample; and 4, GC-JP sample. The images are cropped from loaded lanes of the full-length gel images (Figure S1).

**Figure 5:**
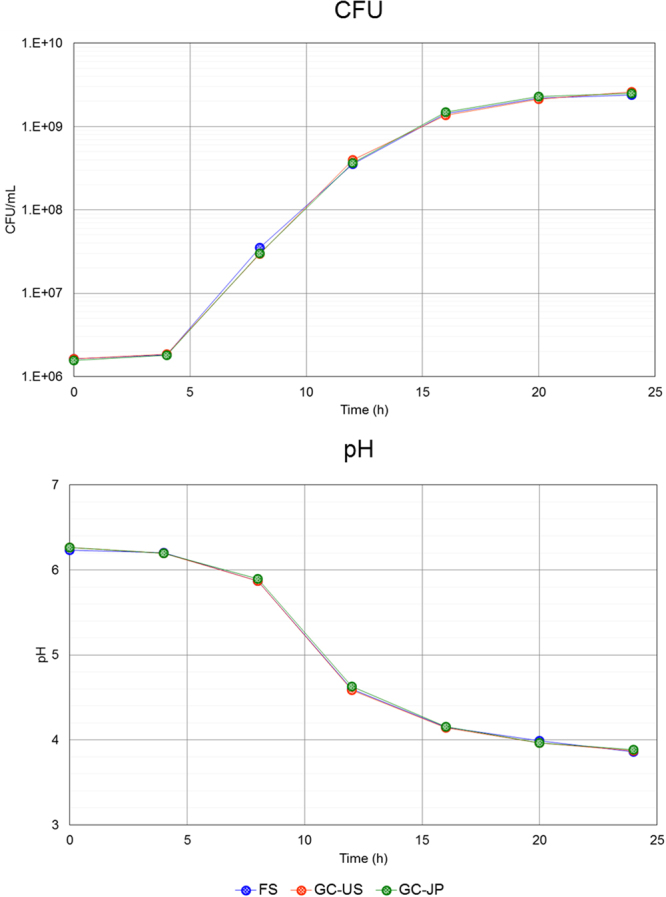
Growth curve and pH profile of the study products in culture medium. Colony-forming units (CFU) of *Lactobacillus casei* strain Shirota and pH values in culture of the flight sample (FS) (blue) and the two ground controls, GC-US (orange) and GC-JP (green), are shown. Each dot indicates a mean value (N = 2).

We examined the band patterns of randomly amplified DNA fragments by using six different primers (Fig. 4 and Figure S1). Randomly amplified polymorphic DNA (RAPD) analysis revealed no genetic modification in the FS compared with baseline (the DNA extracted soon after packaging of the study product), GC-US, or GC-JP. We also conducted whole genome resequencing of the FS, GC-US, GC-JP and baseline samples (Table S1). Sequence variances from the reference LcS sequence (unpublished) in the chromosome as well as in the plasmid pLY101 were evaluated (Figure S2). Profiles of the sequence variant frequency were similar among the study products, and no significant single nucleotide variant was detected. Carbohydrate fermentation properties were not altered by the spaceflight (Table 2 and Figure S3). The growth curve and the pH change profile of LcS in the FS were also similar to those of the ground controls (Fig. 5).

**Table 2:** Results of carbohydrate fermentation testing of the flight sample (FS) and the US and Japanese ground controls (GC-US and GC-JP).

Sugar source	FS	GC-US	GC-JP
Glycerol			
Erythritol			
D-Arabinose			
L-Arabinose			
Ribose	+	+	+
D-Xylose			
L-Xylose			
Adonitol	+	+	+
β-Methyl-xyloside			
Galactose	+	+	+
D-Glucose	+	+	+
D-Fructose	+	+	+
D-Mannose	+	+	+
L-Sorbose	+	+	+
Rhamnose			
Dulcitol			
Inositol			
Mannitol	+	+	+
Sorbitol	+	+	+
α-Methyl-D-mannoside			
α-Methyl-D-glucoside	+	+	+
*N*-Acethyl-glucosamine	+	+	+
Amygdaline	+	+	+
Arbutine	+	+	+
Esculin	+	+	+
Salicin	+	+	+
Cellobiose	+	+	+
Maltose	+	+	+
Lactose	+	+	+
Melibiose			
Saccharose	+	+	+
Treharose	+	+	+
Inulin			
Melezitose			
D-Raffinose			
Amidon			
Glycogen			
Xylitol			
β-Gentiobiose	+	+	+
D-Turanose	+	+	+
D-Lyxose			
D-Tagatose	+	+	+
D-Fucose			
L-Fucose			
D-Arabitol			
L-Arabitol			
Gluconate	w	w	w
2-Keto-gluconate			
5-Keto-gluconate			

Positive; w, weak positive; blank, negative.

### Immune modulation properties

**Figure 6:**
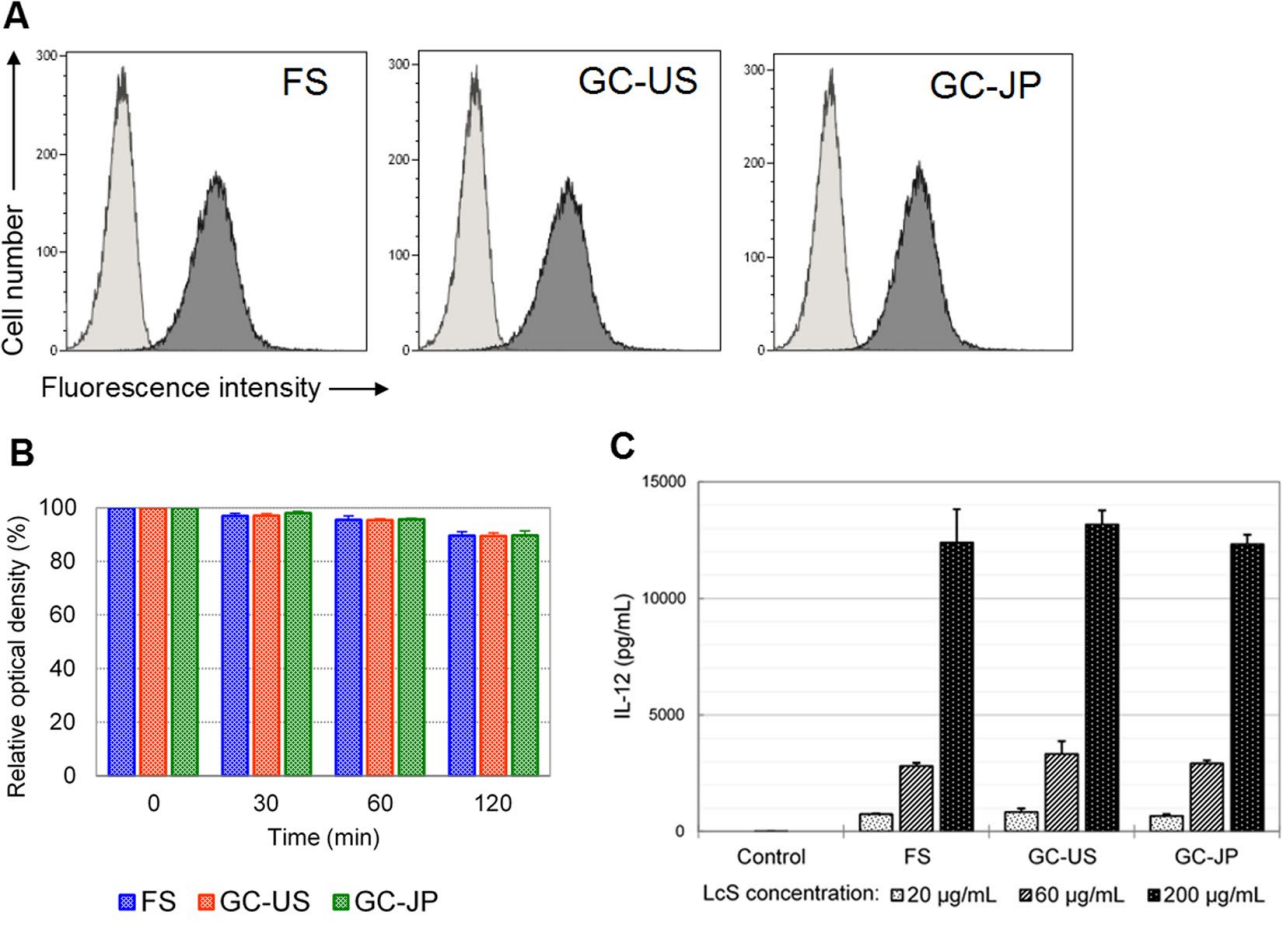
Immune-function-associated analyses. (**A**) Histograms of reactivity to *Lactobacillus casei* strain Shirota–specific monoclonal antibody. Light grey indicates cells without antibody and dark grey indicates cells bound to the antibody. (**B**) Test of resistance to cell-wall digestive enzyme. Relative optical densities in comparison with the initial values are shown as percentages. Each bar chart and error bar indicate mean value ± standard deviation, respectively (N = 3). (**C**) Cytokine production analyses. Each bar chart and error bar indicate mean value ± standard deviation, respectively (N = 3). FS, flight sample; GC-US and GC-JP, the US and Japanese ground controls, respectively.

The cell-wall polysaccharides of LcS and the cell-wall structure, which is resistant to digestion by phagocytes, play important roles in modulation of the host immune system by LcS^25,26^. Therefore, the integrity of the cell-wall polysaccharides was evaluated by analysing reactivity to the monoclonal antibody L8, which is specific to the polysaccharides of LcS^27^, and resistance to intracellular digestion was examined *in vitro* by using *N*-acetylmuramidase^9,25,26^. The microorganisms in the FS reacted with the L8 antibody in the same manner as those in GC-US and GC-JP (Fig. 6A). LcS in all the study products was resistant to the cell-wall digestive enzyme (Fig. 6B). Using the mouse-macrophage-derived cell line J774.1, we evaluated the IL-12-inducing ability of LcS in the FS, GC-US and GC-JP. All samples dose-dependently induced IL-12 at equivalent levels (Fig. 6C).

## Discussion

To the best of our knowledge, no scientific report has been published before regarding the stability of probiotic products on the ISS. This study has thus opened the first door towards the utilisation of live beneficial microorganisms as functional food materials in outer space.

Environmental factors such as temperature and humidity affect the stability of live microorganisms. In addition, there are several factors unique to the inside of the ISS, namely the close confinement, microgravity and space radiation. It is very important to monitor these parameters as far as is possible. Here, actual space radiation dosimetry data were collected as measurable space-specific variables. Also, temperatures during storage of the study product were monitored. We did not collect humidity data, because the study products contained desiccants. The daily irradiation dose to the FS was 0.26 mGy/day, or 0.52 mSv/day. These values are of the same order as those reported previously^28,29^. Environmental temperature on the ISS modules is controlled between 21 and 23 °C on average^30^. The actual temperature data obtained in this study ranged between 20.0 and 24.5 °C. Considering this information collectively, the environmental conditions around the FS could be deemed as typical of those inside the ISS and can be extrapolated to operations in subsequent human studies using astronauts. The temperature conditions of GC-US and GC-JP, at about 22 °C, were also scientifically sound as ground controls for comparison with the FS data.

For probiotics to be effective, it is essential to sustain sufficient numbers of live microorganisms until administration. Over the study period, including during storage at ambient temperatures for approximately 5 months on Earth and in the ISS, the count of live LcS in the FS was maintained at 10^11^ CFU/g powder. One gram of LcS powder is the daily dose for human study subjects (equal to 5 capsules/day). A daily dose of 4 × 10^10^ CFU or more of LcS is preferable for augmentation of immune function^8,10–13^. Our results suggest that the Probiotics Package is capable of maintaining live LcS in sufficient numbers, even on the ISS.

Viability is a good indicator of basic probiotic properties, but viability alone may not be enough to elucidate the stability of LcS during spaceflight. Therefore, we conducted genetic analyses and functional assessments of LcS in the FS *in vitro* and compared the results with those of the ground controls. RAPD analyses and carbohydrate fermentation tests are used frequently to identify bacterial species or strains from genetic and metabolic perspectives. These assessments revealed no differences in the FS compared with GC-US and GC-JP (Table 2 and Fig. 4). In addition, the sequence variant frequency analyses (Figure S2) did not detect any genetic modification in the FS in comparison with the ground controls. The growth pattern, indicating the activity of live microorganisms, did not differ between the study products (Fig. 5). Reactivity to an LcS-specific monoclonal antibody, resistance to a cell-wall digestive enzyme, and IL-12-inducing ability were assessed to evaluated potential immune modulation properties. LcS in the FS, as well as in GC-US and GC-JP, reacted with the monoclonal antibody (Fig. 6A), was resistant to the cell-wall digestive enzyme (Fig. 6B), and induced IL-12 production (Fig. 6C). These results are in line with the fundamental characteristics of LcS^25–27^. Considering all of these results, we concluded that the genetic and functional characteristics of LcS in the Probiotics Package did not alter during storage on the ISS for 1 month.

Microbiological investigations in outer space, mainly using pathogens, suggest that spaceflight may alter the behaviour of microorganisms; for instance, it increases the virulence of *Salmonella typhimurium*^31,32^, promotes biofilm formation by *Pseudomonas aeruginosa*^33^, and reduces the viability of *Staphylococcus epidermidis*^34^. Recent microbiological experiments with simulated microgravity models have yielded controversial differences in the results obtained with aerobic and with anaerobic conditions in terms of the stability of *Lactobacillus acidophilus*^35,36^. All such experiments were conducted with culture media in which the microbes were in an active state. In contrast, the Probiotics Package was devised to minimise the moisture content and activity of LcS until its consumption by crew members. The configuration employed in the Probiotics Package is more suitable than the liquid form of probiotics for avoiding the uncontrollable effects of the ISS environment.

We acknowledge that there are several limitations to interpretation of the study results. The most important issue is the short duration (1 month) of storage on the ISS in our study, because most astronauts stay there for longer: about half a year. The number of resupply vehicles is also limited at the moment; therefore, the practical storage duration of the study product is currently deemed to be a couple of months. Because of limited resources, it was not possible to store the Probiotics Package for longer this time.

We took a ‘macro’ approach by using a series of biochemical assays, a RAPD analysis and a sequence variant frequency analysis, with the primary aim of assessing the direct properties of the probiotic as a food material; we did not address the effect of the outer space environment on the rate of mutations. However, the latter would be of concern to the scientific community, as has been argued in other microbiological experiments in space^34,37^. This will be tested as a part of our future study.

In conclusion, 1 month’s storage of the Probiotics Package on the ISS did not affect the basic properties of LcS as probiotics. The quality of probiotics is maintained well by the product’s configuration.

## Materials and Methods

### Study products

The freeze-dried LcS powder, the LcS capsules, and the Probiotics Packages were manufactured by Yakult Central Institute (YCI; Tokyo, Japan). The LcS capsules were packaged in PTP sheets by API Co., Ltd (Gifu, Japan). Ten capsules were packed in each PTP sheet. Four PTP sheets were then packed in an aluminium-laminated bag containing desiccants. FS, one of the GC-JP samples and one of the GC-US samples contained a Bio PADLES package (JAXA) as well as a temperature data logger. The Bio PADLES package was used to measure space radiation doses to lactobacilli on the ISS or on Earth, including during airfreight between Japan and the US.

### Timeline of study product handling

All dates in this paragraph are in JST. The study products were packaged on 2 December 2015 and kept in cool conditions (refrigeration, and cooler boxes during transport). The FS and GC-US were transported to the US by air, whereas GC-JP stayed in a refrigerator at YCI. On 14 December 2015, the FS was moved to a warehouse at ambient temperature. At about that time, GC-US and GC-JP were transferred into incubators (temperature set at 22 °C) at the National Aeronautics and Space Administration (NASA) Johnson Space Center (JSC; Houston, TX, USA) and at YCI, respectively. After transport to NASA Kennedy Space Center (FL, USA), the FS was stowed in the SpX-8. On 9 April 2016, SpX-8 was launched and docked successfully with the ISS the next day. The FS was then stowed in the Japanese experimental module ‘Kibo’. The FS was brought back into the Dragon spacecraft about 1 month later and returned to Earth on 12 May 2016. After splashing down in the Pacific Ocean, the FS was recovered from the Dragon capsule and kept cool during transport to JSC. Upon arrival at JSC, the FS was transferred into a refrigerator. GC-US and GC-JP were transferred into refrigerators on the day of the splashdown. The FS and GC-US were brought back to Japan by air in cooler boxes, then delivered to YCI on 27 May 2016 for assessment. The temperature data loggers and Bio PADLES packages were sent to JAXA afterwards, for temperature and space radiation dosimetry analyses.

### Space radiation dosimetry

Each Bio PADLES package comprised four CR-39 plastic nuclear track detector plates and seven thermoluminescent dosimeter elements. The dosimetry method used by Bio PADLES is described in previous papers^29,38–41^.

### Enumeration of LcS by culture method and by PMA-qPCR

For culture, 0.1 g of freeze-dried LcS powder in an LcS capsule from each study product was suspended in 9.9 mL of saline and then subjected to 10-fold serial dilutions. The suspensions were incubated aerobically on Plate Count Agar with BCP (Nissui Pharmaceutical Co. Ltd, Tokyo, Japan, for measurements at YCI; HIMEDIA Laboratories Pvt, Ltd, Mumbai, India, for measurements at JSC) at 37 °C for up to 72 h. Incubated plates containing 30 to 300 colonies were used for enumeration.

For PMA-qPCR, 0.1 g of LcS powder was suspended in 9.9 mL of PBS. Then, 200 μL of the suspension was added to 800 μL of PBS. After centrifugation of the suspension at 20,000 *g* at 4 °C for 5 min, the resulting pellet was washed twice. The washed pellet was suspended in 2 μL of 5 mM PMA (Biotium Inc., Fremont, CA, USA), kept on ice for 5 min, and then subjected to photoactivation by LED Crosslinker (Takara Bio Inc., Shiga, Japan) for 10 min. The PMA-treated sample was preserved at −80 °C until DNA extraction. DNA extraction and quantitative PCR were conducted in accordance with previous reports^42^ using LcS-specific primers^43^, namely pLcS-57F, CTCAAAGCCGTG ACGGTC, and pLcS-597R, ACGTGGTGCTAATAATCCTAGTG (5′–3′).

### RAPD analysis

A spatula portion of LcS powder was incubated in MRS medium at 37 °C overnight. After centrifugation (20,400 *g*, 4 °C for 3 min), the pellet was resuspended with DNA extraction buffer. Glass beads and benzyl chloride were added to the suspension, and the mixture was vortexed vigorously using FastPrep (MP Biomedicals LLC, Santa Ana, CA, USA). After denaturing of the proteins by addition of sodium dodecyl sulphate, sodium acetate was added for DNA extraction and the mixture was centrifuged (20,400 *g*, 4 °C for 8 min). The resulting supernatant was subjected to isopropanol precipitation. The DNA collected by centrifugation was suspended in TE buffer. The amplification program was as follows: one cycle at 94 °C for 2 min; five cycles at 94 °C for 30 s, 36 °C for 60 s, and 72 °C for 90 s; 29 cycles at 94 °C for 20 s, 36 °C for 30 s, and 72 °C for 90 s; and one cycle at 72 °C for 3 min. Sequences of the primers (5′–3′) were as follows; p1254, CCGCAGCCAA^44^; p1281, AACGCGCAAC^44^; p1252, GCGGAAATAG^44^; p1280, GAGGACAAAG^44^; pRPIR, GGCGTCGGTT^45^; and pRPICGR, GGCCACGGAA^45^. The amplified DNA fragments were subjected to 1.5% agarose gel electrophoresis.

### Whole genome resequencing and sequence variance analyses

LcS powder was suspended in MRS medium to make 0.1% (w/v) suspension and incubated at 37 °C up to 300 Klett units. After centrifugation twice (10,000 g, 4 °C for 30 minutes and 20,000 g, 4 °C for 5 minutes, respectively), the pellet was resuspended with TE buffer (10 mM Tris-HCl and 1 mM EDTA, pH 8.0). Genomic DNA was prepared using ZR Fungal/Bacterial DNA Mini Prep^TM^ (Zymo Research Corp., Irvine, CA, USA) in accordance with the manufacturer’s instructions. Then, 2.1 μg of the genomic DNA were fragmented to the size of around 800 bp by DNA Shearing system M220 (Covaris, Inc., Woburn, MA, USA). Sequence libraries were prepared using TruSeq DNA PCR-Free Sample Prep LS Kit (Illumina, Inc., San Diego, CA, USA). After denaturation treatment, 15 pM of the libraries were subjected to the paired-end sequencing (250 bp × 2) with MiSeq (Illumina, Inc.).

The read data was imported to CLC Genomics Workbench (CLC Bio, Finlandsgade, Denmark), and sequence trimming was conducted by the following settings; trim using quality scores limit = 0.05; trim ambiguous nucleotides = 2; and discard reads below length = 150. Cleaned sequence reads were mapped to the reference LcS genome assembly (currently not available on public repository because of the proprietary data of Yakult Honsha Co., Ltd; summary of the genome analysis is described in the GRAS notice document^46^). Variant calling was performed using SAMtools^47^. Sequence variant frequency was evaluated on the position where the following conditions were satisfied; depth for the chromosome >sequence coverage ×1/3; depth for the plasmid >3× sequence coverage ×1/3; and SP (Phred-scaled strand bias P-value) <13. The variant frequency higher than 50% is deemed to be a significant single nucleotide variant.

### Carbohydrate fermentation test

A spatula portion of LcS powder was incubated in MRS medium at 37 °C overnight. A portion of the culture was incubated in new MRS medium at 37 °C overnight again. After centrifugation of the suspension at 1,660 *g* at 4 °C for 20 min, the resulting pellet was suspended in API 50 CHL medium (SYSMEX bioMérieux Co., Ltd, Tokyo, Japan). An API 50 CH strip was used in accordance with the manufacturer’s instructions to evaluate the ability of the suspension to ferment 49 carbohydrates.

### Growth pattern analysis

All the LcS powder in one capsule was suspended in 10 mL of MRS medium. The suspension was further diluted 20 times in MRS medium, and the diluted suspension was incubated in new MRS medium at 1% (v/v) at 37 °C for up to 24 h. Culture samples were collected every 4 h, and the LcS were enumerated as colony-forming units upon culture on MRS agar medium (37 °C, 48 to 72 h). We also measured the pH of the cultured samples with a pH meter.

### Assessment of reactivity to LcS-specific antibody

LcS powder was suspended in PBS at 2 mg/mL and kept for 30 min at room temperature. The supernatant was collected as an LcS suspension. Four hundred microlitres of LcS suspension was mixed with 600 µL of PBS. After centrifugation at 10,000 *g* at 4 °C for 5 min, the pellet was subjected to reactivity assessment against the LcS-specific monoclonal antibody L8^27^, using 100 µL of a 10-times dilution of its hybridoma cell culture supernatant. After incubation at 4 °C overnight, the cells were washed twice with PBS and then incubated with 100 µL of an FITC-labelled mouse immunoglobulin light-chain antibody (clone RMK-45, BioLegend, San Diego, CA, USA; 200 times dilution) at room temperature for 20 min. After being washed with PBS, the cells were resuspended in PBS and analysed by using a Gallios flow cytometer (Beckman Coulter Inc., Fullerton, CA, USA).

### Test for resistance to cell-wall digestive enzyme

M-1 cell-wall digestive enzyme (*N*-acetylmuramidase SG, Seikagaku Co. Ltd, Tokyo, Japan) was added to the same LcS suspension as was prepared for the analysis of antibody reactivity. The final concentration was 50 µg/mL, and the suspension was incubated at 37 °C for up to 120 min. At time points of 0, 30, 60 and 120 min, 200 µL of the reaction solution was collected and heated at 100 °C for 5 min to terminate the enzyme reaction. Then, 50 µL of 10% sodium dodecyl sulphate solution was added and the mixture was mixed well. The optical density of the solution was measured at a wavelength of 600 nm, and a relative value (%) was calculated by comparison with the initial value.

### Cytokine production analysis

The same LcS suspension as was prepared for the analysis of antibody reactivity was added at final concentrations of 20, 60 or 200 µg/mL to 10% FCS/RPMI 1640 medium containing 50 U/mL penicillin and 50 µg/mL streptomycin. The mixture was then incubated with the J774.1 cell line (1 × 10^5^ cells/200 µL in each well) in a 96-well culture plate for 24 h at 37 °C. The supernatant was then subjected to enzyme-linked immunosorbent assay to measure IL-12p40, as reported previously^25^.

## Electronic supplementary material

Table S1, Figure S1, Figure S2 and Figure S3

## References

[1] Crucian BE (2014). Plasma cytokine concentrations indicate that *in vivo* hormonal regulation of immunity is altered during long-duration spaceflight. J. Interferon Cytokine Res..

[2] Stowe RP, Sams CF, Pierson DL (2011). Adrenocortical and immune responses following short- and long-duration spaceflight. Aviat. Space Environ. Med..

[3] Guéguinou N (2009). Could spaceflight-associated immune system weakening preclude the expansion of human presence beyond Earth’s orbit?. J. Leukoc. Biol..

[4] Crucian BE, Stowe RP, Pierson DL, Sams CF (2008). Immune system dysregulation following short- vs long-duration spaceflight. Aviat. Space Environ. Med..

[5] Ryokova MP, Antropova EN, Larina IM, Morukov BV (2008). Humoral and cellular immunity in cosmonauts after the ISS missions. Acta Astronautica..

[6] Konstantinova IV, Ryokova MP, Lesnyak AT, Antropova EA (1993). Immune changes during long-duration missions. J. Leukoc. Biol..

[7] Food and Agriculture Organization of the United Nations/World Health Organization (FAO/WHO). Health and nutrition properties of probiotics in food including powder milk with live lactic acid bacteria. http://www.fao.org/3/a-a0512e.pdf (2001).

[8] Reale M (2012). Daily intake of *Lactobacillus casei* Shirota increases natural killer cell activity in smokers. Br. J. Nutr..

[9] Shida K, Suzuki T, Kiyoshima-Shibata J, Shimada S, Nanno M (2006). Essential roles of monocytes in stimulating human peripheral blood mononuclear cells with *Lactobacillus casei* to produce cytokines and augment natural killer cell activity. Clin. Vaccine Immunol..

[10] Takeda K (2006). Interleukin-12 is involved in the enhancement of human natural killer cell activity by *Lactobacillus casei* Shirota. Clin. Exp. Immunol..

[11] Morimoto K, Takeshita T, Nanno M, Tokudome S, Nakayama K (2005). Modulation of natural killer cell activity by supplementation of fermented milk containing *Lactobacillus casei* in habitual smokers. Prev. Med..

[12] Nagao F, Nakayama M, Muto T, Okumura K (2000). Effects of a fermented milk drink containing *Lactobacillus casei* strain Shirota on the immune system in healthy human subjects. Biosci. Biotechnol. Biochem..

[13] Shida K (2017). Daily intake of fermented milk with *Lactobacillus casei* strain Shirota reduces the incidence and duration of upper respiratory tract infections in healthy middle-aged office workers. Eur. J. Nutr..

[14] Mai TT, Hop DV, Anh TT, Lam NT (2017). Recovery of *Lactobacillus casei* strain Shirota (LcS) from the intestine of healthy Vietnamese adults after intake of fermented milk. Asia Pac. J. Clin. Nutr..

[15] Wang R (2015). Survival of *Lactobacillus casei* strain Shirota in the intestines of healthy Chinese adults. Microbiol. Immunol..

[16] Tilley L (2014). A probiotic fermented milk drink containing *Lactobacillus casei* strain Shirota improves stool consistency of subjects with hard stools. Int. J. Probiotics Prebiotics..

[17] Tiengrim S, Leelaporn A, Manatsathit S, Thamlikitkul V (2012). Viability of *Lactobacillus casei* strain Shirota (LcS) from feces of Thai healthy subjects regularly taking milk product containing LcS. J. Med. Assoc. Thai..

[18] Sakai T (2010). M-RTLV agar, a novel selective medium to distinguish *Lactobacillus casei* and *Lactobacillus paracasei* from *Lactobacillus rhamnosus*. Int. J. Food Microbiol..

[19] Matsumoto K (2006). The effects of a probiotic milk product containing *Lactobacillus casei* strain Shirota on the defecation frequency and the intestinal microflora of sub-optimal health state volunteers: a randomized placebo-controlled cross-over study. Biosci. Microflora..

[20] Nagata S (2016). The effectiveness of *Lactobacillus* beverages in controlling infections among the residents of an aged care facility: a randomized placebo-controlled double-blind trial. Ann. Nutr. Metab..

[21] Wang C (2015). Intestinal microbiota profiles of healthy pre-school and school-age children and effects of probiotic supplementation. Ann. Nutr. Metab..

[22] Nagata S (2011). Effect of the continuous intake of probiotic-fermented milk containing *Lactobacillus casei* strain Shirota on fever in a mass outbreak of norovirus gastroenteritis and the faecal microflora in a health service facility for the aged. Br. J. Nutr..

[23] U.S. Food and Drug Administration. Agency Response Letter GRAS Notice No. GRN 000429. https://wayback.archive-it.org/7993/20171031024628/https://www.fda.gov/Food/IngredientsPackagingLabeling/GRAS/NoticeInventory/ucm335746.htm (2012).

[24] Fujimoto J, Tanigawa K, Kudo Y, Makino H, Watanabe K (2011). Identification and quantification of viable *Bifidobacterium breve* strain Yakult in human faeces by using strain-specific primers and propidium monoazide. J. Appl. Microbiol..

[25] Yasuda E, Serata M, Sako T (2008). Suppressive effect on activation of macrophages by *Lactobacillus casei* strain Shirota genes determining the synthesis of cell wall-associated polysaccharides. Appl. Environ. Microbiol..

[26] Shida K, Kiyoshima-Shibata J, Nagaoka M, Watanabe K, Nanno M (2006). Induction of interleukin-12 by *Lactobacillus* strains having a rigid cell wall resistant to intracellular digestion. J. Dairy Sci..

[27] Yuki N (1999). Survival of a probiotic, *Lactobacillus casei* strain Shirota, in the gastrointestinal tract: selective isolation from faeces and identification using monoclonal antibodies. Int. J. Food Microbiol..

[28] Wakayama S (2017). Healthy offspring from freeze-dried mouse spermatozoa held on the International Space Station for 9 months. Proc. Natl. Acad. Sci..

[29] Nagamatsu A (2006). Development of the space radiation dosimetry system ‘PADLES’. KEK Proc..

[30] Thirsk R, Kuipers A, Mukai C, Williams D (2009). The space-flight environment: the International Space Station and beyond. CMAJ..

[31] Wilson JW (2008). Media ion composition controls regulatory and virulence response of *Salmonella* in spaceflight. PLoS One..

[32] Wilson JW (2007). Space flight alters bacterial gene expression and virulence and reveals a role for global regulator Hfq. Proc. Natl. Acad. Sci..

[33] Kim W (2013). Spaceflight promotes biofilm formation by *Pseudomonas aeruginosa*. PLoS One..

[34] Fajardo-Cavazos P, Nicholson WL (2016). Cultivation of *Staphylococcus epidermidis* in the human spaceflight environment leads to alterations in the frequency and spectrum of spontaneous rifampicin-resistance mutations in the *rpoB* gene. Front. Microbiol..

[35] Castro-Wallace S, Stahl S, Voorhies A, Lorenzi H, Douglas GL (2017). Response of *Lactobacillus acidophilus* ATCC 4356 to low-shear modeled microgravity. Acta Astronautica..

[36] Shao D (2017). Simulated microgravity affects some biological characteristics of *Lactobacillus acidophilus*. Appl. Microbiol. Biotechnol..

[37] Moeller R, Reitz G, Nicholson WL, The PROTECT Team, & Horneck, G (2012). Mutagenesis in bacterial spores exposed to space and simulated martian conditions: data from the EXPOSE-E spaceflight experiment PROTECT. Astrobiology..

[38] Tawara H (2008). Measurement of linear energy transfer distribution with antioxidant doped CR-39 correcting for the dip angle dependence of track formation sensitivity. Jpn. J. Appl. Phys..

[39] Nagamatsu A (2008). Space radiation dosimetry in low Earth orbit by passive and integrating dosimeter ‘PADRES’. KEK Proc..

[40] Nagamatsu A (2011). Radiation damage to HDTV camera CCDs on board the International Space Station. Radiation Measurements..

[41] Tawara H (2011). Characteristics of Mg2SiO4:Tb (TLD-MSO) relevant for space radiation dosimetry. Radiation Measurements..

[42] Matsuki T (2004). Quantitative PCR with 16S rRNA-gene-targeted species-specific primers for analysis of human intestinal bifidobacteria. Appl. Environ. Microbiol..

[43] Fujimoto J, Matsuki T, Sasamoto M, Tomii Y, Watanabe K (2008). Identification and quantification of *Lactobacillus casei* strain Shirota in human feces with strain-specific primers derived from randomly amplified polymorphic DNA. Int. J. Food Microbiol..

[44] Akopyanz N, Bukanov NO, Westblom TU, Kresovich S, Berg DE (1992). DNA diversity among clinical isolates of *Helicobacter pylori* detected by PCR-based RAPD fingerprinting. Nucleic Acids Res..

[45] Endo A, Okada S (2006). *Oenococcus kitaharae* sp. nov., a non-acidophilic and non-malolactic-fermenting oenococcus isolated from a composting distilled shochu residue. Int. J. Syst. Evol. Microbiol..

[46] JHeimbach LLC. Generally Recognized as Safe (GRAS) Determination for the Use of *Lactobacillus casei* Strain Shirota As a Food Ingredient. http://wayback.archive-it.org/7993/20171031055001/https://www.fda.gov/downloads/Food/IngredientsPackagingLabeling/GRAS/NoticeInventory/ucm309143.pdf (2012).

[47] Li H (2009). The Sequence Alignment/Map format and SAMtools. Bioinformatics..

