# Supplementary material for: Probiotics into outer space: feasibility assessments of encapsulated freeze-dried probiotics during 1 month’s storage on the International Space Station
Source: Sci Rep. 2018 Jul 16;8:10687. doi: 10.1038/s41598-018-29094-2 (PMC6048169; doi:10.1038/s41598-018-29094-2)
Supplement: Supplementary file 1 — Table S1, Figure S1, Figure S2 and Figure S3 [file 41598_2018_29094_MOESM1_ESM.pdf]

Probiotics into outer space: feasibility assessments of encapsulated freeze-dried probiotics during 1 month's storage on the International Space Station

Takafumi Sakai[1], Yasuhiro Moteki[1], Takuya Takahashi[1], Kan Shida[1], Mayumi Kiwaki[1], Yasuhisa Shimakawa[1], Akihisa Matsui[1], Osamu Chonan[1], Kazuya Morikawa[2,3], Toshiko Ohta[2,3], Hiroshi Ohshima[3], Satoshi Furukawa[3]

[1]Yakult Central Institute, Yakult Honsha Co., Ltd, Tokyo 186-8650, Japan, [2] Division of Biomedical Science, Faculty of Medicine, University of Tsukuba, Ibaraki 305-8575, Japan, [3]Japan Aerospace Exploration Agency, Ibaraki 305-8505, Japan. Correspondence and requests for materials should be addressed to T.S. (takafumi-sakai@yakult.co.jp).

Supplementary Table S1. Average numbers of sequence coverage.

Sample	Sequence coverage
Baseline_1	127.9
Baseline_2	129.0
FS_1	126.0
FS_2	134.9
GC-US_1	126.9
GC-US_2	143.5
GC-JP_1	123.4
GC-JP_2	151.7

FS, flight sample; GC-US and GC-JP, the US and Japanese ground controls, respectively. The genomic DNA extracted soon after packaging the study product was used for the analysis of baseline. Estimated plasmid copy number (three copies) was taken into consideration for the analysis.

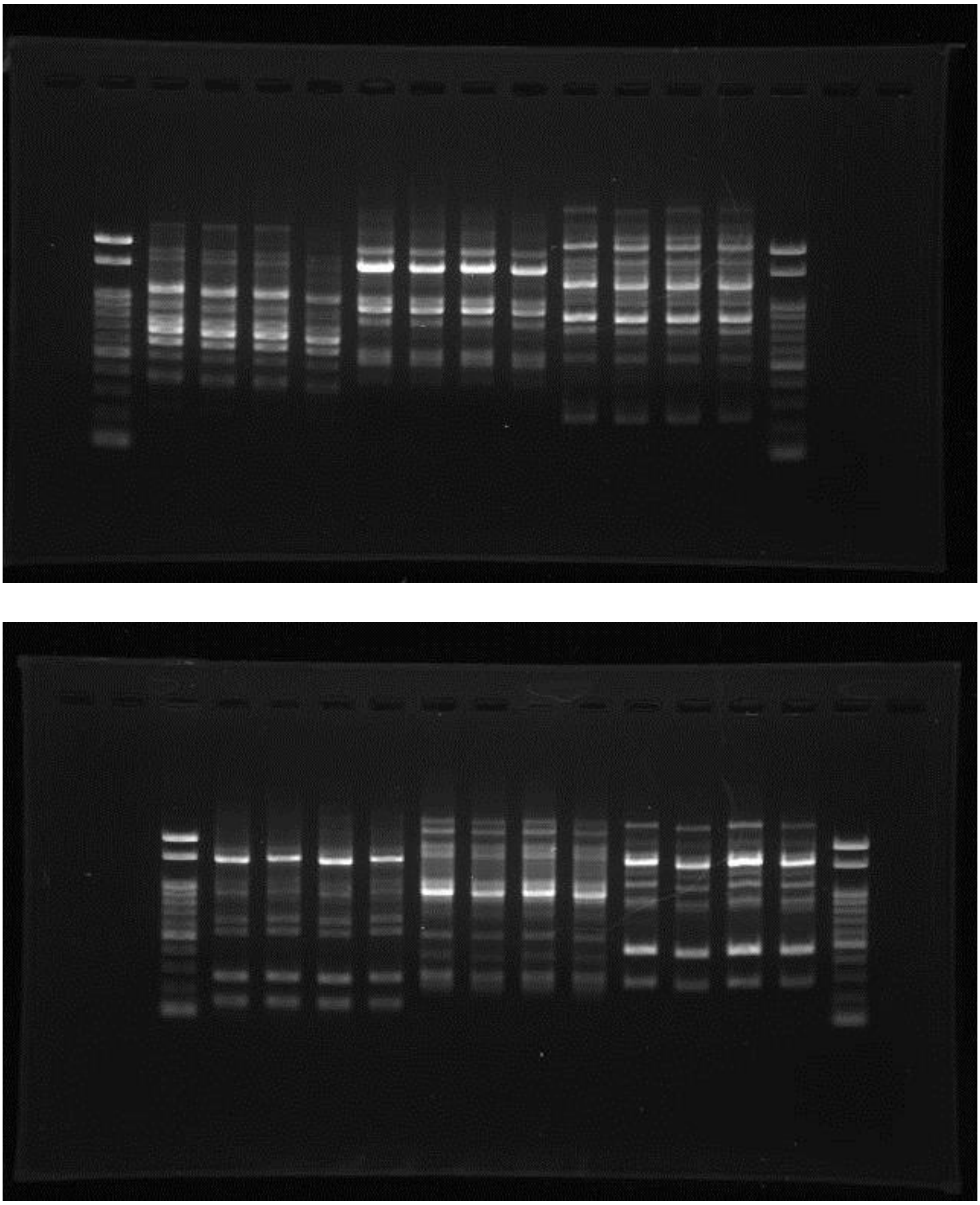

Supplementary Figure S1. Full-length gel images of the randomly amplified polymorphic DNA analysis profiles shown in Figure 4. Upper image; profiles with p1254, p1281 and p1252 primers, lower image; profiles with p1280, pRPIR and pRPICGR primers.

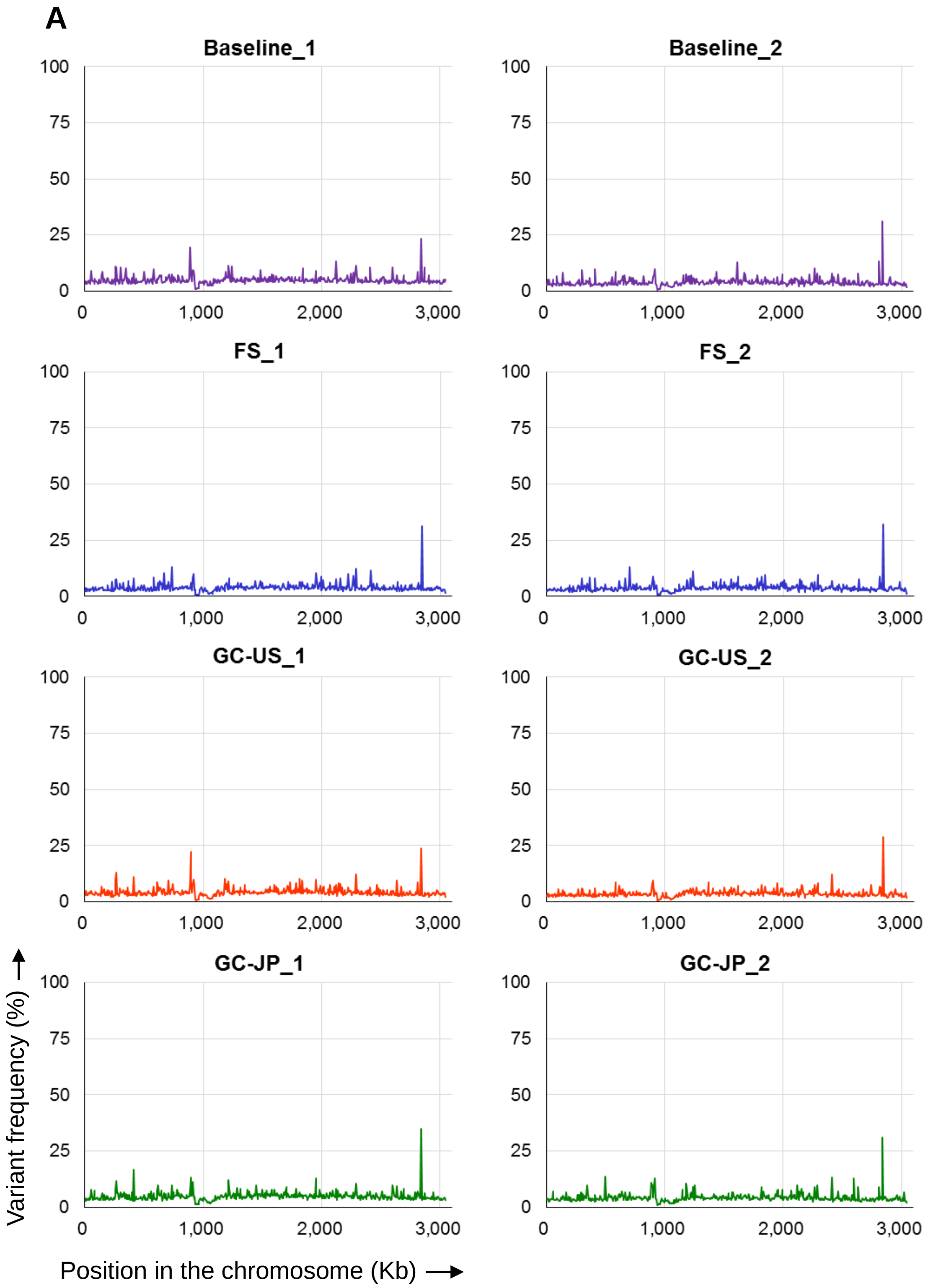

Supplementary Figure S2. Profiles of sequence variant frequency analysis of the baseline (using the genomic DNA extracted soon after packaging the study product, purple), the flight sample (FS, blue), and the two ground controls, GC-US (orange) and GC-JP (green) in duplicate (N =2). The variant frequencies on the chromosome (A) and on the plasmid pLY101 (B) are shown. Estimated plasmid copy number (three copies) was taken into consideration the analysis.

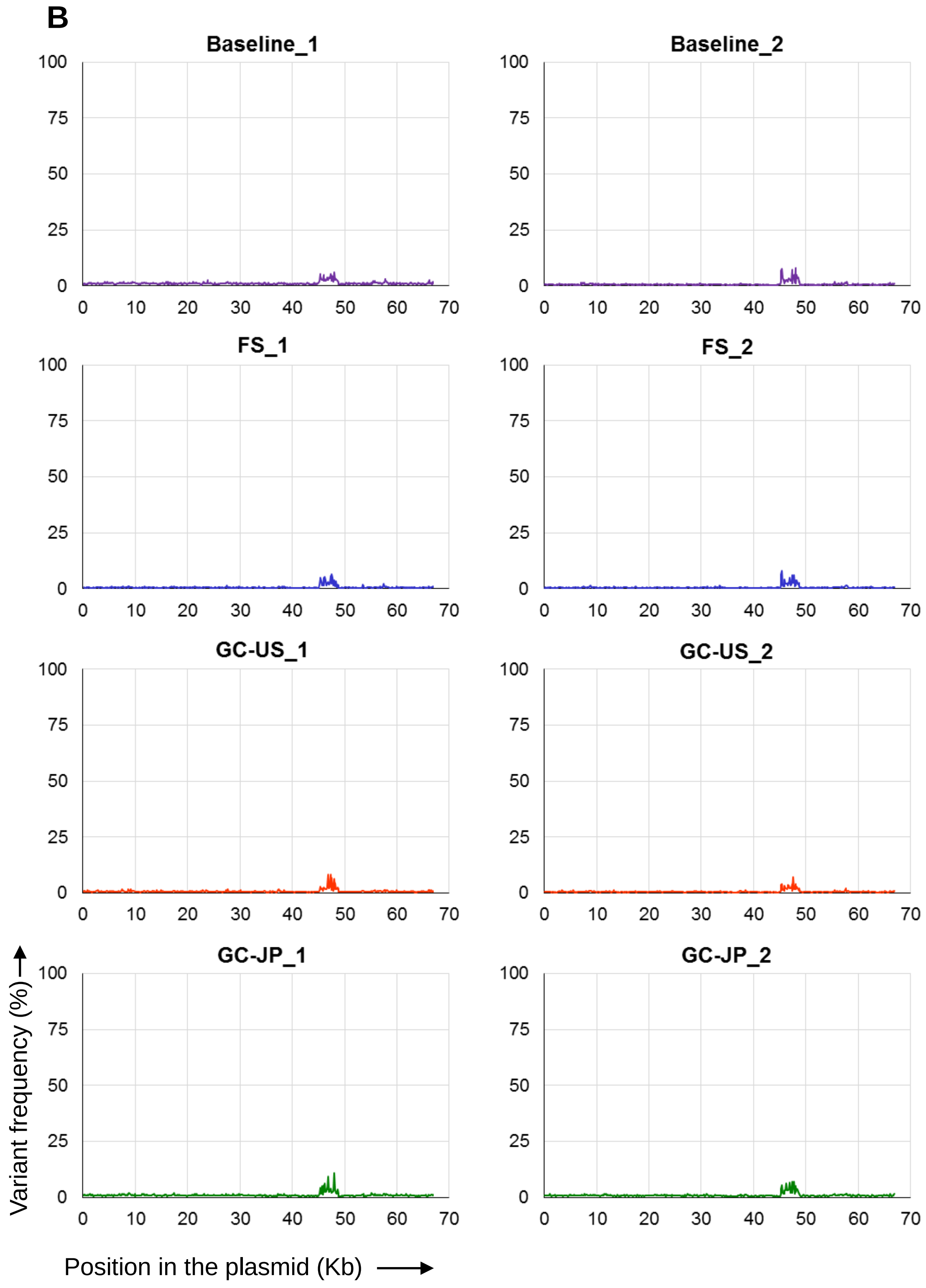

Supplementary Figure S2 (continue).

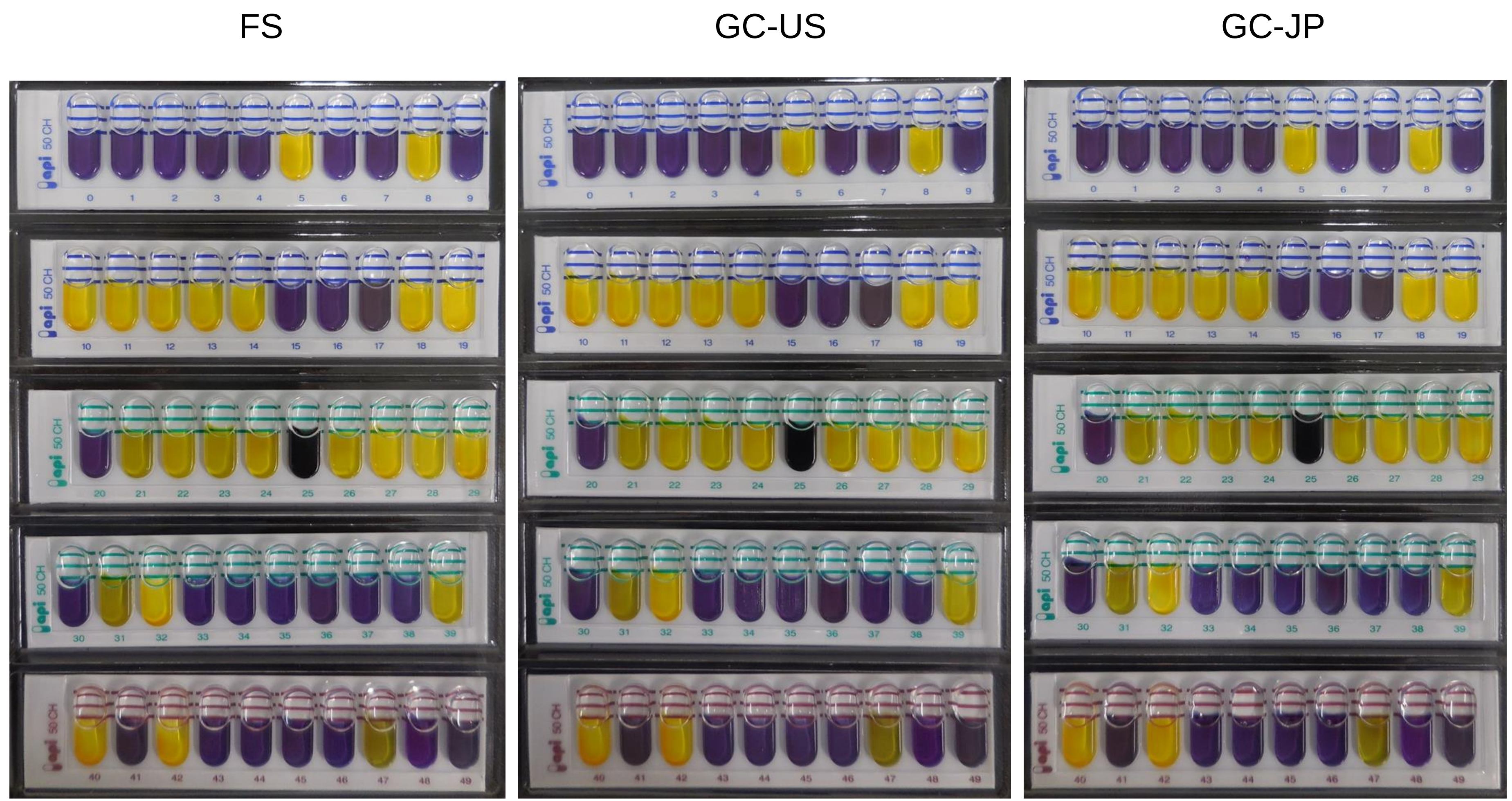

Supplementary Figure S3. API 50 CH strips after incubation. FS, flight sample; GC-US and GC-JP, the US and Japanese ground controls, respectively.
